# Prophylactic Ureteral Catheterization for Preventing Ureteral Injury in Colorectal Cancer Surgery

**DOI:** 10.3390/jcm14124123

**Published:** 2025-06-11

**Authors:** Shinobu Ohnuma, Keigo Kanehara, Yukihiro Sato, Tomoyuki Ono, Megumi Murakami, Taiki Kajiwara, Hideyuki Suzuki, Hideaki Karasawa, Kazuhiro Watanabe, Naoki Kawamorita, Akihiro Ito, Takashi Kamei, Michiaki Unno

**Affiliations:** 1 Department of Surgery, Tohoku University Graduate School of Medicine, Sendai 980-8574, Miyagi, Japan; 2 Department of Urology, Tohoku University Graduate School of Medicine, Sendai 980-8574, Miyagi, Japan

**Keywords:** prophylactic ureteral catheterization, iatrogenic ureteral injury, colorectal cancer

## Abstract

**Background/Objective:** Iatrogenic ureteral injury is a rare but serious complication of colorectal cancer surgery. Although prophylactic ureteral catheterization (PUC) is used to facilitate intraoperative ureter identification and reduce the risk of ureteral injury, its efficacy is debated. We aimed to evaluate the clinical utility and outcomes of PUC in colorectal cancer surgery. **Methods**: This retrospective study included 42 patients who underwent PUC before colorectal cancer surgery at the Tohoku University Hospital between February 2010 and September 2024. Preoperative ureteral stents were inserted via cystoscopy under general anesthesia. Patient demographics, surgical techniques, indications for catheterization, and post-procedural complications were reviewed. **Results**: PUC was most frequently performed in patients with left-sided colorectal cancer (61.9%) and local recurrence of rectal cancer (31%). Ureteral catheterization was indicated in patients with a history of pelvic surgery (47.6%) or tumor proximity to the ureter (26.2%). Open surgery was performed in 90.5% of the cases, whereas robotic surgery with fluorescent ureteral catheters was used in selected patients. No intraoperative ureteral injury was observed in the stent group. Catheter-related complications, including hematuria (14.3%) and urinary tract infections (9.5%), were minor and resolved before discharge. **Conclusions**: PUC may be beneficial in patients with a history of pelvic surgery or local recurrence of rectal cancer, in whom the risk of ureteral injury is inherently higher.

## 1. Introduction

Colorectal cancer (CRC) is one of the leading malignancies worldwide in terms of incidence and mortality. According to the 2022 estimates, approximately 1,926,136 new cases of CRC were diagnosed globally, accounting for approximately 9.6% of all cancer cases. Additionally, CRC was responsible for approximately 903,853 deaths, representing 9.3% of all cancer-related deaths. It was the third leading cause of cancer mortality, following lung and breast cancer [1]. In Japan, CRC also remains one of the most prevalent malignancies. In 2022, the number of deaths from CRC reached 53,088, with 28,099 deaths in men and 24,989 in women. Among all cancer-related deaths in Japan, CRC was the second leading cause of cancer mortality, following lung cancer [2].

The primary principle of CRC treatment is the resection of the tumor. In addition to conventional open surgery, minimally invasive surgery, such as laparoscopic or robotic-assisted techniques, has become increasingly widespread, leading to recent changes in surgical approaches for CRC. Laparoscopic surgery has been reported to achieve superior short-term outcomes compared to open surgery while demonstrating equivalent long-term oncological outcomes [3,4,5,6]. Furthermore, robotic-assisted surgery offers additional advantages beyond those of laparoscopic surgery. It provides a stable, tremor-free, high-definition, 3D, magnified view, enabling delicate dissection while clearly visualizing the anatomical structures. Additionally, the surgical instruments (scissors and graspers) with their enhanced degrees of freedom enable more refined and flexible movements, along with tremor filtration, facilitating highly precise surgical maneuvers [7,8,9].

Although minimally invasive surgery has begun to be applied to advanced tumors, such as T3 and T4 lesions [10], for locally advanced and recurrent CRC, extended resection via open surgery is still required [11,12]. Surgical procedures for such advanced and recurrent CRC have been reported to be associated with a complication rate of 24–78%, including pelvic abscess, wound infections, bleeding, and bowel obstruction [13,14].

Iatrogenic ureteral injury is a complication of CRC surgery, with an incidence ranging from 0.2% to 2.0% [15,16]. Although rare, it can lead to severe postoperative complications. Prophylactic ureteral catheterization (PUC) has been employed as a preventive measure to facilitate the intraoperative identification of the ureter, thereby reducing the risk of injury. Additionally, it aids in the early detection of ureteral injuries [17,18]. Despite evidence suggesting the potential protective effect of PUC, its efficacy remains controversial. PUC itself may cause complications such as hematuria, reflux, acute kidney injury, and even ureteral injury despite catheter placement [19,20,21]. Furthermore, some studies have indicated that PUC does not significantly reduce the incidence of ureteral injury [22,23]. A consensus regarding the indications for ureteral stent placement to prevent iatrogenic ureteral injury has not yet been established. In this study, we present our institutional experience with PUC for CRC surgery and evaluate its clinical utility and outcomes.

## 2. Materials and Methods

This study included 42 patients who underwent surgery for primary or locally recurrent CRC following PUC at Tohoku University Hospital, Sendai, Japan, between February 2010 and September 2024. Benign diseases, such as Crohn’s disease, ulcerative colitis, and diverticulitis, as well as colorectal malignancies other than adenocarcinoma, gynecological tumors, and urological tumors, were excluded from this study. Patients were selected for preoperative ureteral catheterization based on their history of pelvic surgery by laparotomy, history of pelvic radiotherapy, tumor proximity to the ureter, inflammation in the pelvic cavity, and morbid obesity through a preoperative review conference conducted by colorectal surgeons. Preoperative computed tomography scans were performed to evaluate the stage of CRC and to assess the status of urinary organs, including the presence of hydronephrosis or ureteral dilation. Urine output was monitored intraoperatively and postoperatively, and renal function was assessed on postoperative days 1, 3, and 7.

The term iatrogenic was defined as the accidental occurrence of a ureteral lesion. The ureteral injury was defined as damage to the ureter associated with the surgery and classified into the following categories: intraoperative ureteral injury included direct trauma (transection, perforation, or laceration), thermal injury (heat-related damage caused by electrocautery or energy devices), ischemic damage resulting from ligation or suturing, and obstruction caused by clips or staplers. Postoperative ureteral injury was defined as ureteral stricture, obstruction, or urinary leakage occurring after surgery. Cases in which the ureter was intentionally resected to achieve complete resection of the tumor were excluded from the analysis. Nine board-certified colorectal surgeons performed open surgery. Three colorectal surgeons trained in robotic surgery performed all of the robotic procedures.

Ureteral stents were inserted preoperatively by urologists under general anesthesia using cystoscopy. A standardized stent size was employed for all patients. Specifically, a catheter was inserted approximately 20 cm into the ureter from the ureteral orifice. For open surgery, a Ureteral Catheter TigerTail® Flexible Open Tip Polyurethane 5 Fr (Bard Medical Division, Covington, GA, USA) was used, whereas for robotic surgery, a near-infrared ray catheter (Cardinal Health, Tokyo, Japan), a fluorescent ureteral catheter, was employed. Patient characteristics, including sex, age, diagnosis, surgical approach, laterality of ureteral catheterization (unilateral or bilateral), and indications for ureteral catheterization were examined based on the patient’s medical chart and operative records. Complications associated with ureteral catheterization were also assessed. The variables were collected from the patients’ medical records, and the data were managed in the form of an Excel spreadsheet. Statistical analyses were performed using JMP Pro 17.2 (SAS Institute Japan Ltd., Tokyo, Japan).

Informed consent was obtained for CRC surgery and for PUC. Patients were informed of the potential complications and the possibility of prolonged surgical time associated with PUC.

This retrospective, single-institution study was approved by the Ethics Committee of Tohoku University (Approval No. 2020-1-1055).

## 3. Results

**Figure 1:**
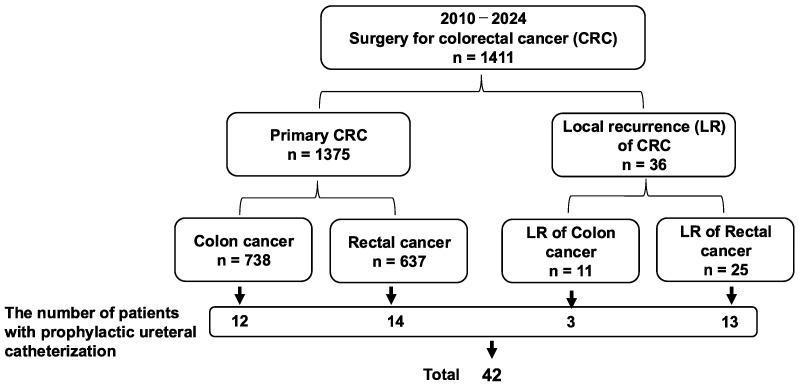
Flowchart of patient selection in this study. CRC: colorectal cancer, LR: local recurrence.

During the study period, from February 2010 to September 2024, 42 patients with primary or recurrent CRC underwent surgery following PUC. Among them, 19 were male and 23 were female, with a median age of 58.5 years (range: 30–83 years). During the same period, 1411 CRC resections were performed, including 738 colon cancer cases, 637 rectal cancer cases, 25 cases of locally recurrent rectal cancer, and 11 cases of locally recurrent colon cancer, resulting in a ureteral stent insertion rate of 3.0% (42/1411) (Figure 1).

The surgical indications are summarized in Table 1, with primary rectal cancer being the most common (33.3%), followed by locally recurrent rectal cancer (31.0%). The surgical procedures performed are detailed in Table 2, with abdominoperineal resection accounting for 35.7%, low anterior resection for 21.4%, and sigmoid colectomy for 19.0% of cases. Regarding the surgical approach, 38 patients (90.5%) underwent open surgery, whereas 4 patients (9.5%) underwent robotic surgery.

The indications for PUC are shown in Table 3, with previous pelvic surgery by laparotomy (47.6%) being the most common reason, followed by tumor proximity to the ureter (26.2%), history of radiotherapy (19.0%), inflammation in the pelvic cavity (16.7%), and morbid obesity (2.4%). Catheter insertion was bilateral in 60% of the cases and unilateral in 40%. Among the 17 patients who underwent unilateral catheterization, 14 had left-sided placement and 3 had right-sided placement. The median time required for catheterization was 8 min (range: 4–21 min) for unilateral and 13 min (range: 5–27 min) for bilateral placement, as shown in Table 4. In all patients, the ureteral catheter was removed at the end of the surgery.

Complications associated with ureteral catheterization, including macroscopic hematuria (14.3%), dysuria (12.0%), and urinary tract infection (9.5%), are summarized in Table 5. No ureteral injuries associated with ureteral catheterization or intraoperative ureteral injuries were observed.

## 4. Discussion

Iatrogenic ureteral injury is a severe complication of CRC surgery. As a retroperitoneal organ, the ureter is particularly susceptible to injury during the mobilization of the colon and rectum. This risk increases in cases involving reoperation or bulky tumors. The most crucial factor in preventing ureteral injury is ensuring proper identification of the ureter. PUC has been used to identify the ureter and prevent ureteral injury [17,18]. In open surgery, tactile feedback aids in identifying the ureter, whereas in laparoscopic and robotic procedures, the visual recognition of the ureter plays a key role in minimizing injury. Furthermore, even when ureteral injury occurs, immediate recognition allows for prompt intraoperative repair of the ureter.

This study evaluated the clinical significance of PUC. Among all CRC surgeries performed, ureteral stents were inserted in 3.0% of the cases. Although the decision to perform PUC often depends on the surgeon’s experience and preference, it is more frequently performed in patients with left-sided CRC, including rectal or sigmoid colon cancer, as well as local recurrence of rectal cancer. Furthermore, PUC was indicated for patients with a history of pelvic surgery or in cases in which the tumor was in close proximity to the ureter, that is, in cases with a high risk of ureteral injury. Consequently, in our study, the rate of PUC was higher in patients with local recurrence of rectal cancer.

**Figure 2:**
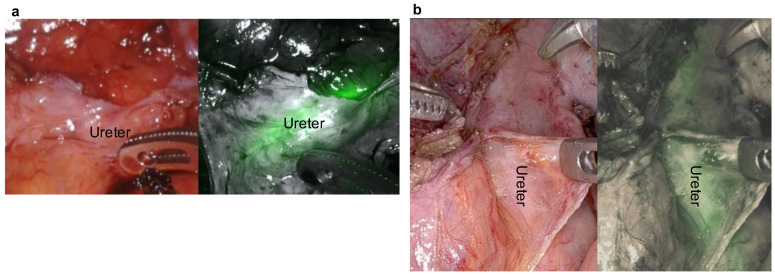
Identification of the ureter with the help of a fluorescent ureteral stent (**a**) in a rectal cancer patient with a body mass index of 44 kg/m^2^ and (**b**) in a patient with lateral lymph node metastasis.

Patients with a history of pelvic surgery or those with local recurrence of rectal cancer have traditionally undergone open surgery. The reason for selecting open surgery in most cases in this study is based on the characteristics of the patient population. Many of the included cases had a history of pelvic surgery or local invasion resulting from advanced disease, making laparoscopic or robot-assisted surgery technically challenging and associated with higher risks. In such cases, open surgery was often considered more appropriate from a safety perspective. Moreover, even in open surgery, there were many instances where the ureter was difficult to recognize because of adhesions, inflammation, or tumor invasion. Ensuring adequate identification is crucial not only in laparoscopic or robot-assisted surgery but in all types of surgical procedures. However, robotic surgery, which offers better visualization and maneuverability, is being increasingly used for these patients. Unlike open surgery, robotic surgery, in which haptic feedback is not available, relies entirely on visual feedback. Therefore, fluorescent ureteral stents have been used in robotic procedures to enhance ureteral identification. In our experience, robotic surgery was performed following the placement of a fluorescent ureteral stent in a patient with rectal cancer and a body mass index of 44 kg/m^2^. Patients with rectal cancer and morbid obesity have a heightened risk of misidentification of the dissection plane, which increases the likelihood of ureteral injury. Placement of the ureteral stent facilitated visualization of the ureter, enabling safe and accurate dissection (Figure 2a). Additionally, in cases of local recurrence of rectal cancer (lateral pelvic lymph node metastasis), ureteral stents facilitate the identification and dissection of the ureter, allowing for the safe removal of metastatic lymph nodes under robotic assistance (Figure 2b). Fluorescent ureteral stents contain dyes with optical properties similar to those of indocyanine green enabling near-infrared cameras to visualize the ureteral anatomy. Numerous studies have reported the benefits of fluorescence-guided ureteral navigation in minimally invasive surgery [24,25]. These findings suggest that fluorescent ureteral stents can aid in preventing ureteral injury by improving dissection accuracy.

In this study, no cases of intraoperative ureteral injury were observed in the patients who underwent PUC. In contrast, among the 1369 patients who did not receive stents during the same period, two cases of ureteral injury (0.15%) were reported. One case was a patient with T4b appendiceal cancer, where the tumor was in close proximity to the ureter, and the other was a patient with locally recurrent sigmoid colon cancer. The intraoperatively detected injury in the former case was repaired using uretero-ureteral anastomosis, whereas the postoperatively detected injury in the latter case necessitated nephrostomy placement, leading to prolonged hospitalization. The pelvic region is the area most susceptible to iatrogenic injuries in CRC surgery, with rectal cancer cases demonstrating the highest rates of ureteral injuries (0.7%) [26]. As previously reported, ureteral catheterization appears to be beneficial in patients in whom ureteral identification is challenging, such as those with a history of pelvic surgery, recurrent CRC, or T4b tumors [27,28,29].

PUC is associated with a certain degree of invasiveness, including the risk of procedure-related complications such as infection and/or trauma, as well as increased surgical and anesthesia times, thereby imposing an additional burden on patients. However, PUC serves as an adjunctive measure to facilitate intraoperative identification of the ureters and may significantly contribute to the prevention of irreversible ureteral injury, particularly in cases with a history of pelvic surgery or local recurrence of rectal cancer. This, in turn, leads to a reduction in long-term complications such as ureteral strictures, renal dysfunction, and the need for reoperation. Moreover, when comparing the incidence of mild to moderate PUC-related complications (e.g., infection or hematuria) with the risk of severe long-term complications associated with ureteral injury, we believe that the balance of risks and benefits favors the use of PUC. The mean extension of operative time associated with PUC placement was also limited, and we consider that the benefit of enhanced safety outweighs the slight decrease in surgical efficiency. Furthermore, the complications associated with ureteral catheterization in our study were generally minor, with macroscopic hematuria and urinary tract infection classified as Clavien–Dindo grade ≤2, all of which were resolved before discharge. Although urinary dysfunction was observed, it remains unclear whether this was directly attributable to ureteral catheterization or was instead a consequence of autonomic nerve disruption resulting from surgical manipulation of the pelvic cavity.

While intraoperative ureteral injury can often be avoided during CRC surgery with PUS, there remains a risk of postoperative ureteral stricture (US). Moretto et al. reported a US incidence of 0.3–4.9% in patients who underwent endoscopic treatment for urolithiasis [30]. Furthermore, factors influencing iatrogenic US in endoscopic urolithiasis treatment have been investigated. These include Stone-related factors such as impacted stones, Procedural factors including inadequate visualization or poor surgical technique, Instrumentation and access-related factors like the use of large-caliber ureteral access sheaths or excessive ureteral dilation, and patient-related factors such as anatomical abnormalities or a history of prior ureteral manipulation [31]. Given these considerations, there is a potential for postoperative ureteral stricture even in CRC surgery with PUS. Therefore, minimizing mechanical trauma and selecting appropriate instruments are crucial strategies for preventing iatrogenic US following CRC surgery with PUS.

In daily clinical practice, PUC may typically be performed by urologists. However, there is growing interest in having surgeons perform PUC themselves. For example, the potential benefits of surgeon-performed PUC may increase flexibility in surgical scheduling. The procedure can be performed without relying on the availability of a urologist, allowing for smoother surgical workflows, which may reduce anesthesia and total operative time. It is especially advantageous in emergency settings. Since some fields, such as gynecology and urogynecology, have integrated PUC into residency, it may be worth considering incorporating PUC into surgical residency program as well [32].

PUC has been reported to be effective in preventing ureteral injury and facilitating its intraoperative detection. However, it may also be associated with prolonged operative time and an increased risk of postoperative complications [19,20,21,33]. Additionally, previous studies have suggested that ureteral stent placement does not contribute to an overall cost reduction. Although ureteral injury reduction and intraoperative detection may be achieved, these benefits do not necessarily offset the hospital costs associated with stent placement during colorectal surgery [34]. Moreover, there is a lack of robust statistical evidence demonstrating that ureteral stenting significantly reduces the incidence of ureteral injury [22,23]. Therefore, there is no established consensus regarding indications for PUC. The European Urological Association recommends a case-by-case approach to ureteral catheterization, considering patient-specific risk factors and the surgeon’s experience [35].

As an alternative approach, ureteral identification can be achieved by injecting indocyanine green directly into the ureter, allowing for its visualization and localization with a near-infrared camera [36]. Furthermore, ultrasound-guided ureteral identification [37] has also been reported. These approaches are advantageous in that they do not require catheterization and are free from the risk of infection. However, their effectiveness is highly dependent on the surgeon’s skill level and the availability of specialized equipment at each institution. Particularly in complex pelvic surgeries or in patients with a history of abdominal surgeries, these methods may not sufficiently guarantee a reduced risk of ureteral injury. Preoperative computed tomography urography is a useful tool for understanding ureteral anatomy and may provide sufficient safety in cases with a low-risk profile [38]. However, in high-risk cases—such as those with severe adhesions, a history of pelvic operations, or bulky tumors—which we considered as indications for PUC, we believe that PUC offers superior safety compared to these alternative methods and remains a highly valuable strategy.

The future perspective for PUC in colorectal surgery is shaped by several factors:

Advancement in Minimally Invasive Surgery: As minimally invasive techniques, such as robotic-assisted surgery and laparoscopic approaches, continue to improve, the precision of identifying and protecting the ureters during colorectal surgery will also improve. In particular, robotic surgery offers enhanced visualization and greater dexterity, which can reduce the need for PUC. However, PUC may remain a useful tool in cases where there is a higher risk of ureteral injury, especially in challenging pelvic surgeries.

Risk Stratification and Personalized Approach: With increasing capabilities for patient-specific risk assessments, PUC may become more tailored to individual patients. For example, patients with advanced-stage CRC (such as T4) or those with a history of pelvic surgeries and local recurrence of rectal cancer are at greater risk of ureteral injury. In these cases, PUC could be more frequently utilized. Future research may lead to better preoperative identification of patients who would benefit most from this intervention, thus reducing unnecessary use and associated complications.

Minimizing Complications: Although PUC is a useful technique to prevent ureteral injury, ureteral stenting is associated with certain complications, including urinary tract infections, hematuria, and kidney damage. Novel methods are currently being developed to enhance intraoperative ureteral identification without stent placement. One example is the use of fluorescent dyes intravenously administered and excreted in urine, which allow for real-time visualization of the ureter during surgery [39]. For instance, sodium fluorescein, used in ophthalmic diagnostics and venography, is excreted in urine and has been reported to allow visualization of the ureter [40]. Additionally, a new near-infrared fluorescent dye, UreterGlow, can be injected systemically but is primarily excreted through the renal system, enabling ureter visualization in pigs using a near-infrared fluorescence camera [41]. Both have been reported in animal studies, and the validation on humans is still needed. However, such innovations hold promise for reducing operative time and minimizing the complications associated with ureteral catheterization.

This study had some limitations. This was a single-institution retrospective study with a relatively small sample size, and the overall incidence of ureteral injury was low. Therefore, the true effect of PUC in colorectal surgery remains unclear. Additionally, as this was not a comparative study, the decision to perform PUC was influenced by the surgeon’s experience and preference, introducing a potential bias. Prospective multi-institutional studies are warranted to comprehensively evaluate the clinical utility of ureteral stenting in colorectal surgery.

## 5. Conclusions

In conclusion, with advances in anatomical understanding and standardization of surgical techniques, PUC may not be routinely required for primary CRC surgery. However, in challenging cases, such as those with a history of pelvic surgery or local recurrence of rectal cancer, PUC may be useful in preventing ureteral injury.

## Figures and Tables

**Table 1:** Indications for surgery.

Indications	No. of Patients (%)
Colon cancer *	12 (28.6)
Rectal cancer	14 (33.3)
Local recurrence of colon cancer	3 (7.1)
Local recurrence of rectal cancer	13 (31.0)

* All tumors were located at the sigmoid colon.

**Table 2:** Number of procedures (%).

Procedures	No. of Patients (%)
Tumor resection	7 (16.7)
Left colectomy	2 (4.8)
Sigmoid colectomy	8 (19.0)
LAR	9 (21.4)
APR	15 (35.7)
TPE	1 (2.4)

LAR, low anterior resection; APR, abdominal perineal resection; TPE, total pelvic exenteration. Robotic approach was performed for 4 patients (tumor resection: 1, LAR: 2, APR: 1).

**Table 3:** Indication of ureteral catheterization.

Indications	No. of Patients (%)
History of pelvic surgery by laparotomy	24 (47.6)
Tumor proximity to ureter	11 (26.2)
History of radiotherapy	8 (19.0)
Inflammation in pelvic cavity	7 (16.7)
Morbid obesity	1 (2.4)

**Table 4:** Side of catheterization and time required.

Side of Catheterization	Unilateral	Bilateral
No. of patients (%)	17 (40) (R: 3, L: 14)	25 (60)
Time (min, range)	8 (4–21)	13 (5–27)

R, right; L, left.

**Table 5:** Complications of ureteral catheterization.

Indications	No. of Patients (%)
Hematuria	6 (14.3)
Dysuria	5 (12.0)
Urinary tract infection	4 (9.5)
Hydronephrosis	1 (2.4)
Ureteral injury	0 (0)

## Data Availability

The raw data supporting the conclusions of this article will be made available by the authors on request.

## Abbreviations

The following abbreviations are used in this manuscript:

APR	Abdominal Perineal Resection
CRC	Colorectal cancer
LAR	Low anterior resection
PUC	Prophylactic ureteral catheterization
TPE	Total Pelvic Exenteration
